# Microbiota dysbiosis and barrier dysfunction in inflammatory bowel disease and colorectal cancers: exploring a common ground hypothesis

**DOI:** 10.1186/s12929-018-0483-8

**Published:** 2018-11-09

**Authors:** Linda Chia-Hui Yu

**Affiliations:** Graduate Institute of Physiology, National Taiwan University College of Medicine, Suite 1020, #1 Jen-Ai Rd. Sec. 1, Taipei, 100 Taiwan, Republic of China

**Keywords:** Colitis, colorectal cancers, intestinal dysbiosis, barrier function, epithelial permeability, bacterial internalization

## Abstract

Inflammatory bowel disease (IBD) is a multifactorial disease which arises as a result of the interaction of genetic, environmental, barrier and microbial factors leading to chronic inflammation in the intestine. Patients with IBD had a higher risk of developing colorectal carcinoma (CRC), of which the subset was classified as colitis-associated cancers. Genetic polymorphism of innate immune receptors had long been considered a major risk factor for IBD, and the mutations were also recently observed in CRC. Altered microbial composition (termed microbiota dybiosis) and dysfunctional gut barrier manifested by epithelial hyperpermeability and high amount of mucosa-associated bacteria were observed in IBD and CRC patients. The findings suggested that aberrant immune responses to penetrating commensal microbes may play key roles in fueling disease progression. Accumulative evidence demonstrated that mucosa-associated bacteria harbored colitogenic and protumoral properties in experimental models, supporting an active role of bacteria as pathobionts (commensal-derived opportunistic pathogens). Nevertheless, the host factors involved in bacterial dysbiosis and conversion mechanisms from lumen-dwelling commensals to mucosal pathobionts remain unclear. Based on the observation of gut leakiness in patients and the evidence of epithelial hyperpermeability prior to the onset of mucosal histopathology in colitic animals, it was postulated that the epithelial barrier dysfunction associated with mucosal enrichment of specific bacterial strains may predispose the shift to disease-associated microbiota. The speculation of leaky gut as an initiating factor for microbiota dysbiosis that eventually led to pathological consequences was proposed as the “common ground hypothesis”, which will be highlighted in this review. Overall, the understanding of the core interplay between gut microbiota and epithelial barriers at early subclinical phases will shed light to novel therapeutic strategies to manage chronic inflammatory disorders and colitis-associated cancers.

## Introduction

Human intestine harbors approximately 3.8 × 10^13^ bacteria, with over 1000 species found in a cohort [1]. Bacteria also habitat the skin, oral and nasal cavity, and vagina; however, the bacterial counts in extraintestinal organs are no more than 10^12^ [1, 2]. Along with the large amount of bacteria, other microorganisms including virus, archaea, and fungi inhabits the gastrointestinal tract and are collectively defined as the gut microbiota [3]. Keeping in mind that the number of gut bacteria is the same order as human cells and the bacterial genes outnumber human genes by 10- to 100- fold, a symbiotic relationship is maintained between the host and the lumen-confined microbes in a healthy state [4]. Recent evidence indicated that altered microbial communities (termed “microbiota dysbiosis”) and intestinal barrier impairment are associated with the development of a number of chronic inflammatory disorders and systemic diseases [5–7]. These included inflammatory bowel disease (IBD), celiac disease, multiple sclerosis, rheumatoid arthritis, ankylosing spondylitis, psoriasis, type 2 diabetes, allergic diseases, cardiovascular and neurodegenerative diseases, and cancers [8–13]. An incoming speculation of common factors involved in the pathogenesis of chronic polygenic disorders has been proposed as the “common ground hypothesis”, which placed microbiota dysbiosis and leaky gut in the core mechanisms of a wide array of diseases.

The breach of mucosal barrier may result in unlimited passages of microbes to lamina propria and systemic bloodstream, which could overturn immune tolerance to hyperactivation in the body. The epithelial barrier defects accompanied by an altered microbial community were observed in patients and experimental models of chronic and acute intestinal diseases, such as IBD (Crohn’s disease (CD) and ulcerative colitis (UC)) [14–17], celiac disease[18–22], bowel obstruction [23–25], and gastrointestinal (GI) infection [26–29]. IBD is a multifactorial disease of unclear etiology, which arises as a result of the interaction of genetic, environmental, barrier and microbial factors leading to immunological responses and chronic inflammation in the intestine. Patients with IBD had a higher risk of developing colorectal carcinoma (CRC) in later life [30]. As genetic polymorphisms of innate immune receptors (such as nucleotide-binding oligomerization domain (NOD) 2/CARD15 and toll-like receptor (TLR) 4 [31–35]) are considered major risk factors for IBD development, aberrant immune response to host own commensal microbiota was considered to play key roles in fueling the progression of inflammatory diseases. Recent evidence demonstrated that immune-related gene mutations were also observed in CRC patients, including polymorphism in TLRs and ATG16L1 (an autophagy gene for control of immune responses to virus and bacteria) [36–38]. Experimental models provided evidence that aberrant epithelial innate immune responses were involved in the pathogenesis of colitis and tumor development [39–43], further supporting a link between microbe, inflammation and cancers.

The purpose of the review is to summarize the evidence of bacterial dysbiosis and barrier dysfunction in patients and experimental models of IBD and CRC, and to discuss the “common ground hypothesis” to explain abnormal host-microbe interactions underlying disease pathogenesis. Lastly, this review offers further speculation on the mechanisms of mucosal enrichment and conversion of commensal-derived pathobionts in the context of inflammation and cancers.

## Microbiota dysbiosis and mucosa-associated bacteria in chronic inflammation

Microbiota dysbiosis is characterized by microbial population, diversity, spatial, or number change in the human body [9, 43]. Stool samples are often used as surrogates for the intestinal microbial contents because it is relatively easy to collect in clinical laboratories. Distinct fecal microbial communities were found between IBD patients and healthy control subjects [44–46]. An average of 25% less microbial richness was found in IBD patients compared to healthy individuals [47–49]. The reduction of microbial diversity with relative abundance or paucity of specific bacterial taxa was widely reported in IBD patients. However, a large variation of fecal bacterial composition in IBD patients was documented in the literatures [50, 51].

An inter-individual variability was readily noted in the fecal microbiota of healthy subjects. Although over one thousand bacterial species were identified in a cohort study with mainly four phyla (Bacteroidetes, Firmicutes, Proteobacteria and Actinobacteria), it should be emphasized that each person harbors around 160 species and that only 30-40 species as the bulk of microbiota are shared among individuals [48, 52, 53]. Studies with Crohn’s patients have shown that *Enterobacteriaceae* family [54, 55], and *Fusobacterium* and *Enterococcus faecalis* [56] were significantly increased in the fecal samples compared to those of healthy subjects. Lower bifidobacterial populations and reduction of butyrate-producing bacteria (such as *Faecalibacterium*, *Eubacterium, Roseburia, Lachnospiraceae* and *Ruminococcaceae*) were found in fecal samples of patients with CD and UC [55, 57–59]. Despite variable results were documented, a reduction of fecal bacterial richness were commonly reported in patients with CD and UC [60–63]. This suggests that maybe fewer species could be making up the majority of a disease-associated microbial population.

While a general consensus exists that altered gut microbiota composition is associated with IBD, a direct causal relationship remains debatable in humans. The uncertainty of causation or correlation is partly due to the fact that stool samples are collected at one single time point in patients (after the diagnosis of IBD) and in healthy subjects without the disorder. Other confounding factors include the dietary habits and life style in individuals, and the use of antibiotics and immunotherapy in patients. Hence, the timing of bacteria dysbiosis relative to disease onset is hard to decipher in humans even by studies of pediatric cohorts [64–66]. The cause-effect relationship of microbiota dysbiosis and chronic inflammatory disorders relied mainly on data of experimental models.

Accumulating evidence indicated that mucosa-associated bacteria are different from fecal microbial population, and may better reflect regional changes in gut microbes at mucosal surfaces at sites of inflammation [50, 53]. In healthy states, indigenous symbiotic bacteria mostly reside in the intestinal lumen which are separated from the epithelial cells by inner firm mucus layers [67], and are not in direct contact with the epithelial cells in physiological conditions [68, 69]. Nevertheless, high densities of mucosa-associated bacteria were reported in IBD patients [64, 65, 70], and were suspected to play a more dominant role than fecal microbiota in promoting gut inflammation. A recent study demonstrated that microbiota obtained from IBD patients from a greater mass of biofilm containing bacteria and extracellular matrix compared to that of healthy controls [71]. Moreover, higher invasiveness of IBD biofilms in a model of human intestinal epithelia was observed compared to healthy control biofilms, demonstrating a more virulent phenotype of microbiota in IBD patients [71].

The enrichment of *Enterobacteriaceae*, *Bacteroides/Prevotella*, *Veillonellaceae, and Fusobacteriaceae* were reported in ileal and colonic biopsies of new-onset treatment-naïve pediatric patients with CD and UC [64–66]. Other studies showed the abundance of the *Escherichia coli* in tissue biopsies of Crohn’s patients [55, 60, 72–74]. In addition, adherent-invasive *E.coli* (AIEC) was found in the ileal lesions of Crohn’s disease patients [72, 75]. Moreover, a high amount of adherent *Bacteroides fragilis* was found in the mucosal biofilm in patients with IBD [64]. Presence of *B. fragilis* and enterotoxigenic *B. fragilis* (ETBF) was found in the stool and biopsy specimens of healthy individuals, but significantly higher toxin genes were detected in UC patients [76–78]. Furthermore, *Enterococcus* strains with adherent and biofilm-forming ability were isolated from tissue biopsies of IBD patients [79]. Taken together, abundance of mucosa-associated bacteria is correlated to gut inflammation.

The role of gut microbiota in colitis development was confirmed by using animal models. Germ-free mice displayed minimal inflammation or delayed onset of chemically and genetically induced colitis (e.g. IL-2(-/-) and IL-10(-/-)) compared to the conventionally raised animals [80–84]. However, higher mortality was seen in germ-free than conventional mice after giving dextran sulfate sodium (DSS) due to massive gut epithelial injury [82, 83]. The seemingly paradoxical phenomenon could be explained by the lack of immune maturation and/or tolerance as well as the impairment of epithelial turnover (which is dependent on commensal colonization) in germ-free intestine [85–87]. With this said, germ-free models provided clear evidence that intestinal bacteria are crucial for the development of colitis. Other studies using co-housing and fecal transplantation experiments demonstrated the existence of “disease-predisposing microbiota” or “pathobionts” (an opportunistic bacteria derived from commensals) in the fecal microbiota [88, 89]. The animal experiments supported that intestinal bacteria played a disease-predisposing role in colitis development.

Recent studies by using monoassociation and inoculation experiments have helped teased out the roles of single strains of colitis-associated bacteria, and provided valuable information in addition to the overall dysbiotic microbiota. The gut bacterial species documented with pro-inflammatory roles are discussed in the following sections along with the underlying colitogenic mechanisms.

### *Escherichia coli*

High levels of mucosa-associated bacteria with adherence and invasive ability were isolated from Crohn’s disease patients [72, 75]. Oral inoculation of Crohn’s associated AIEC (LF82 strain), but not the human laboratory *E.coli* K-12, resulted in severe colitis in transgenic mice overexpressing human carcinoemcryonic antigen adhesion molecule 6 (CEACAM6, a receptor to type 1 pili or fimbriae) [90]. In contrast, AIEC did not colonize nor induce colitis in wild type mice [90]. The colitogenic activity of AIEC was dependent on type 1 pili expression as bacteria deleted of the *fimH* gene failed to induce mucosal inflammation [90].

There are evidence indicated that virulence factors other than fimbriae may be crucial for the colitogenic effects. It is noteworthy that the fimH protein sequence of *E.coli* K-12 strain showed high degree of homology (97%) to the LF82, and it only differed from LF82 by variations at residues Ala-48, Ser-91, and Asn-99 [91]. Moreover, the adherence and invasive ability of fim-mutants of LF82 was restored to wild type levels by transforming a *fim* operon derived from *E. coli* K-12 to the mutant. The finding suggested that the fimbriae synthesized by K-12 also possess adherence properties despite of inability of promoting inflammation. In contrast, a non-invasive laboratory *E.coli* strain JM109 transformed with *fim* operons derived from LF82 or K-12 strains did not gain invasive properties, suggesting that although fimbriae-mediated adherence may facilitate bacterial invasion but is insufficient to cause translocation by itself [91]. Additional mechanisms of Crohn’s associated AIEC related to its colitogenic ability included higher bacterial survival and replication inside macrophages and induction of proinflammatory cyclooxygenase (COX)-2 expression from macrophages [92, 93]. Recent data also showed that AIEC LF82 strain is capable of long term intracellular survival in gut epithelial cells by suppressing autophagy [94–96], which could contribute to long-term infection.

Other studies showed that monoassociation of nonpathogenic *E.coli* and *Enterococcus faecalis* to gnotobiotic IL-10(-/-) mice induced inflammation in the cecum and distal colon, respectively [81]. Dual-association of the two commensal bacteria in gnotobiotic IL-10(-/-) induces aggressive pancolitis and duodenal inflammation [97, 98]. The findings demonstrated that commensal bacteria isolated from healthy subjects could be colitogenic when monoassociated in mice with genetic deficiency but not in wild type mice, suggesting that opportunistic commensals may turn into pathobionts in genetically-predisposed hosts.

### *Bacteroides* subspecies

Commensal *Bacteroides* spp., such as *B. fragils* and *B. vultagus,* have been reported to modulate colitis development. Abundance of enterotoxigenic *B. fragilis* (ETBF) was detected in the stool and biopsy specimens of UC patients [76–78]. ETBF but not its nontoxigenic strain causes persistent colitis after oral inoculation to wild type mice [99] and a more severe form of inflammation in models of chemically induced colitis [100]. Intestinal permeability was increased and epithelial E-cadherin was cleaved *in vivo* in the ETBF-colonized wild type mice [101]. The enterotoxin produced by *B. fragilis* (also known as fragilysin) acted as a metalloprotease for cleavage of junctional protein and induction of epithelial-derived IL-8 synthesis, which were suggested to be involved in the colitogenic ability [102, 103]. Moreover, gnotobiotic mice monoassociated with three strains of *B. vultagus* isolated from UC patients showed exacerbated cecal inflammation after DSS administration [104], suggesting potential pro-inflammatory ability of the bacteria.

### *Enterococcus* species

Increased colonic inflammation was observed in IL-10(-/-) mice after inoculation or monoassociation with *Enterococcus faecalis* and *E. faecium* [105–107]. The colitogenic characteristics of *E. faecalis* was partly attributed to a bacterial gelatinase which was involved in intestinal barrier impairment and degradation of E-cadherin (a junctional protein) in mouse studies [106]. Moreover, bacterial adherence and penetration to mucosal layers and biofilm formation of *E. faecalis* were dependent on an enterococcal polysaccharide antigen [107]. A cell surface-associated lipoprotein on *E. faecalis* stimulated TLR2-mediated dendritic cell activation and contributes to inflammation [107].

In sum, animal models have provided clear evidence of a disease-predisposing role of certain gut bacteria, yet whether the altered bacterial population is involved in the initiation or perpetuation of intestinal inflammation remains debatable. Moreover, mucosa-associated adherent and invasive bacteria may play a more pathogenic role than fecal microbes in IBD progression. The conversion mechanisms and timing of specific commensal bacteria to turn into invasive or colitogenic pathobionts have yet to be determined. Overall, longitudinal investigation of mucosa-associated bacterial changes that represents a smaller pool of gut microbiota may help elucidate the driver or passenger roles of individual microbes for colitis development.

## Microbiota dybiosis and mucosal biofilms in colon cancers

Colon carcinoma is the second most commonly diagnosed cancer. The majority (60-85%) of CRC is classified as sporadic cancers and around 10-30% is familial or hereditary, stressing the importance of environmental and microbial factors in tumorigenesis [108, 109]. IBD accounts for 1-2% of CRC cases, but the cancer risk in UC patients is 5 times higher than the general population and colitis-associated CRC is more aggressive [110]. The hereditary CRC which accounts for <5% of CRC cases have identifiable germline mutation, such as adenomatous polyposis coli (APC) tumor suppressor gene [109]. Patients with APC gene mutation develop hundreds to thousands of colorectal polyps at young age, of which the disease is termed familial adenomatous polyposis (FAP). The FAP patients had a 100% cumulative risk of progression to CRC by the age of 40 years, If the polyps were left untreated [111, 112]. To date, abundant studies have revealed altered fecal microbiota composition and enrichment of mucosa-associated bacteria in patients with CRC or FAP [113–116].

Recent evidence indicated that mucosa-associated bacterial population may play more dominant roles than fecal microbiota in colon carcinogenesis [116–118]. Overabundance of *E. coli* was noted in tumor biopsies in stage I to IV CRC samples, whereas *Fusobacterium nucleatum* was found in stage IV but not in earlier stages of cancers [119, 120]. A recent report showed that more than 50% of FAP patients harbor colonic biofilm with both *E. coli* and *Bacteroides fragilis* [113]. So far, these bacterial strains have been proposed as protumoral pathobionts based on experimental data of animal models.

The experimental models to investigate the roles of bacteria in colon carcinogenesis included conventionalized, germ-free, and gene-modified animals [121]. Studies of verifying an infectious carcinogen in conventionalized wild type situation would bear more resemblance to the heterogeneous population of human CRC. The benefits and caveats of each of these models are highlighted here. It is worth mentioning that commensal-derived pathobionts usually do not colonize well in a healthy gut with a diversified ecosystem. Many studies with bacterial inoculation experiments in conventionalized animals incorporated an antibiotic pretreatment protocol to overcome colonization resistance. However, the antibiotic regimen and the time frame of bacterial colonization varied in different reports [121]. The value of germ-free models is clearly seen as it would facilitate intestinal colonization or monoassociation of inoculated bacteria in a chronic setting of malignant transformation. Nevertheless, cautions were raised regarding the lack of intestinal and systemic immune maturation and/or tolerance in germ-free animals which might confound data interpretation [85–87]. Gene-modified mice that developed spontaneous colorectal cancers were also utilized to verify the hypothesis of protumoral bacteria, including APC(Min/+) mice [117, 122, 123] and mice deficient of NOD-like receptors [88, 89, 124, 125]. There are criticisms of using gene-modified or immune-deficient mice which already had a distinct gut microbiota as a result of altered host genetics, and the clinical implication may be limited to only subsets of patients. While the research values of germ-free and gene-modified animals are undoubtful, it is still difficult to tease out the temporal order of host abnormality versus bacterial dysbiosis in these models. The potential tumorigenic bacterial strains are discussed below.

### *Escherichia coli*

Despite indication of Crohn’s-associated AIEC triggering intestinal inflammation by using transgenic mice overexpressing human CEACAM6 [90], no direct evidence was shown for the involvement of AIEC in cancer development. The induction of local inflammation by AIEC has been implicated as a link for progression to intestinal malignancy. Another report demonstrated an increase in tumor susceptibility in CEACAM6-transgenic mice after AOM treatment [126], suggesting a role of fimbriae (without specifying the bacterial strains) in colon tumorigenesis.

Clinical studies showed that 40% of mucosa-associated *E.coli* from IBD patients, and 67-86% of mucosa-associated *E. coli* obtained from CRC or diverticulosis specimens harbored the *pks* pathogenicity island encoding genotoxic coilbactin [117, 127]. Inoculation of NC101 strain (a mouse isolate of pks-positive *E.coli*) increased colon inflammation and intestinal crypt proliferation in human CEACAM6-transgenic mice [127], and caused DNA damage in colonocytes and promoted tumor growth in AOM-treated IL-10(-/-) mouse models [117, 123]. Recent data demonstrated that monoassociation of pks-positivie *E. coli* increased the tumor burden in gnotobiotic APC(Min/+) mice and APC(Min/+); IL-10(-/-) mice [128]. Moreover, a clinical isolate CCR20 strain (a pks-positive *E. coli* obtained from human CRC samples) induced cellular senescence and increased tumor burden in AOM-treated IL-10(-/-) mouse models[129, 130]. Furthermore, the human CRC-associated *E.coli* triggered macrophage-derived COX-2 production in vitro in a *pks*-independent manner [93], suggesting a genotoxin-independent, immune-mediated mechanism for the protumoral activity of bacteria.

### Enterotoxigenic *Bacteroides fragilis*

Presence of ETBF was identified in mucosal biopsies of 60% of FAP patients in contrast to 30% in control individuals [113]*.* Higher amount of ETBF and *B. fragilis* toxin were observed in late-stage CRC samples [77, 78, 131]. Previous studies demonstrated that colonization of ETBF but not its non-toxigenic counterparts induced chronic colitis and promoted colon tumorigenesis in APC(Min/+) mice [118, 122]. A number of tumorigenic mechanisms of *B. fragilis* toxin have been proposed. *B. fragilis* toxin triggered an inflammatory protumoral signaling caspase in colonic epithelial cells that caused the recruitment of polymorphonuclear immature myeloid cells to promote colon cancers [132]. Other studies indicated that *B. fragilis* toxin may cause oxidative DNA damage or induce epithelial E-cadherin cleavage for barrier disruption [99, 101, 118]. Moreover, ETBF drives Th17 inflammation and also promoted invasion of pks-positive *E. coli* by causing mucus degradation in AOM-treated wild type mice [113, 122]. The findings indicated that synergistic effects of various strains of bacteria in immunomodulation may be involved in promoting colon tumorigenesis.

### *Fusobacterium nucleatum*

Abundance of *Fusobacterium* DNA was observed in tumor tissues positively associated with poor prognosis in cancer patients [133]. Higher tumor burden was demonstrated in APC(Min/+) mice following inoculation of clinical isolates of *F. nucleatum*, and was associated with activation of TLR4/MyD88/NFκB signaling and recruitment of tumor-infiltrating myeloid cells [116, 119]. One report showed that *F. nucleatum did not induce colitis nor* exacerbated colon inflammation in APC(Min/+) mice [116]. In addition, inoculation of *F. nucleatum* did not aggravate intestinal inflammation nor induce tumors in colitic models of IL-10(-/-) and T-bet(-/-)/Rag2(-/-) mice [116]. The findings indicated that inflammation was not involved in the pathogenesis of *Fusobacteria*-mediated tumor progression.

Virulence factors and invasiveness of *F. nucleatum* have been implicated in promoting colon tumorigenesis. Higher transcript levels of FadA (an adhesin of *F. nucleatum*) was identified in carcinoma samples compared to normal mucosal biopsies or adenoma tissues [134]. Xenograft studies in immunodeficient mice have shown that injection of purified FadA protein into the subcutaneously inoculated sites resulted in larger tumor size [134]. Moreover, the invasive characteristic of *F. nucleatum* has been linked to cancer growth. *In vitro* studies demonstrated that FadA-dependent adherence and invasion of *F. nucleatum* was involved in induction of cell hyperproliferation, and FadA binding to E-cadherin induced nuclear translocation of β-catenin for oncogene transcription in human CRC cell lines [134]. Another study indicated that *F. nucleatum* invasion activated a TLR4/PAK-1 cascade for β-catenin signaling in CRC cell lines [135]. Lastly, FadA also enhanced the *E. coli* invasion in endothelial cell lines by using transwell assays [134, 136], further indicating that interaction between bacteria may be cause pathology to the hosts.

## Gut barrier dysfunction in chronic inflammation

Gut leakiness manifested by epithelial hyperpermeability was long documented in CD [137–139] and UC patients [140–142]. Increased macromolecular flux in the intestine has been suggested as a predictor for inflammatory relapse in IBD patients in remission [143, 144]. Experimental models using chemical-induced colitis or genetic deficient mice which develop spontaneous enterocolitis with higher susceptibility to tumor formation have demonstrated that epithelial barrier dysfunction preceded the onset of mucosal inflammation [145–147]. An elegant study showed that mice expressing a dominant negative N-Cadherin mutant lacking an extracellular domain (loss of endogenous E-cadherin) developed histopathological features of Crohn’s disease by 3 months of age [148], supporting that epithelial barrier disruption was a cause for intestinal inflammation. Other reports documented that inhibition of epithelial hyperpermeability attenuated the colitis severity in animal models, providing further evidence of the cause-and-effect relationship [149, 150]. In sum, the loss of gut barrier integrity is an early event which contributes to chronic inflammation.

**Fig. 1:**
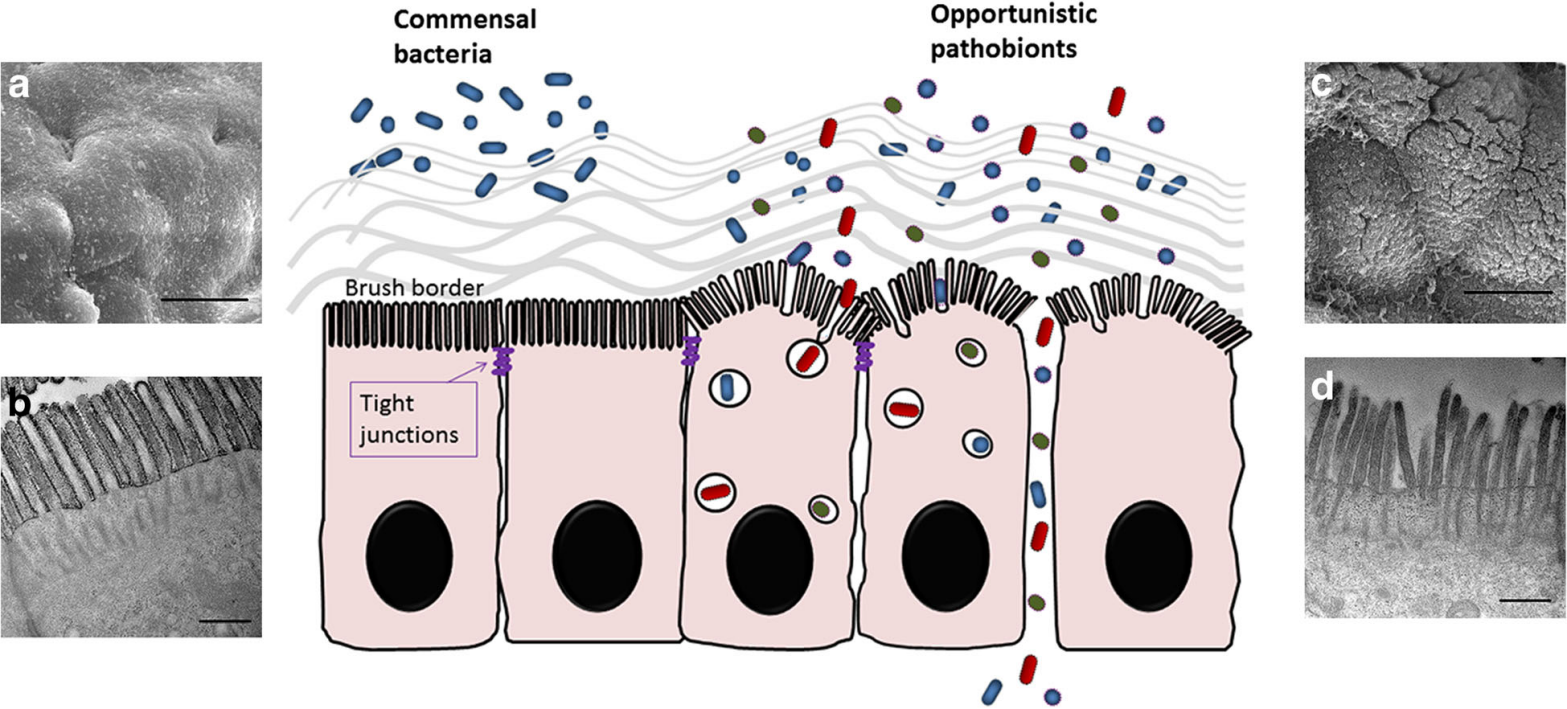
Transcellular and paracellular pathways of epithelial barrier prevents intestinal bacterial influx. Gut barrier is composed of epithelial cells with brush border (BB) as the transcellular barrier, and joined at their apical side by tight junctions (TJs) as the paracellular barrier. The BBs and TJs are physical ultrastructural barriers to prevent influx of commensal bacteria in healthy conditions. Upon epithelial barrier damages such as BB fanning and TJ opening, commensals and pathobionts may gain access to the lamina propria. Photoimages at the left side are (**a**) scanning electron micrographs of the *en face* view and (**b**) transmission electron micrographs of the longitudinal view of the highly organized brush borders in physiological conditions. Photoimages at the right side are (**c**) scanning electron micrographs of the *en face* view and (**d**) transmission electron micrographs of the longitudinal view of the disarrayed brush borders in pathological conditions. (**a**, **c**) Bar = 5 μm; (**b**, **d**) Bar = 0.5 μm

The gut barrier is composed of a single layer of epithelial cells which display densely-packed microvilli (brush border, BB) rooted on terminal webs and are joined at their apical side by tight junctions (TJs) [151–153]. Among the epithelial ultrastructures, the apical BB formed by cytoskeletons separated bacteria from the cellular soma and acted as the transcellular barrier; the TJs formed the most marrow paracellular space and acted as the paracellular barrier. The TJ opening is regulated by activation of myosin light chain kinase (MLCK). In pathological conditions, bacteria may translocate across the epithelial layers through either transcellular or paracellular pathways (Fig. 1).

Both transcellular hyperpermeability (manifested by bacterial internalization to epithelia [154, 155]) and paracellular hyperpermeability (evidenced by abnormal TJ expression and upregulated MLCK activity [156–160]) were noted in mucosal biopsies of patients with CD and UC. While low to negligible amount of bacteria was detected in mucosal tissues of control subjects, presence of mucosal bacteria was found in 83% of colonic specimens from the UC patients, in 56% of the ileal and in 25% of the colonic specimens from the CD patients [65]. Other reports showed 5- and 14-fold higher invasiveness of microbiota biofilms obtained from CD and UC patients, respectively, into a human model of intestinal epithelia, compared to those of healthy control biofilms [71]. Several strains of bacteria, including *E. coli, E. faecalis B. vultagus, Fusobacterium varium* isolated from CD or UC patients were found to invade epithelial cells *in vitro* [107, 155, 161]. Taken together, host barrier defects and microbial invasiveness were both documented in IBD patients.

Other than the transcytotic route, paracellular bacterial influx following TJ disruption was also observed in *in vitro* epithelial cultures [162–166]. However, the timing of two pathways (transcellular versus paracellular) was variable depending on the types of triggers in the context-specific models. To date, longitudinal studies that identify the time points of transcellular and paracellular barrier defects in animal models of colitis are still lacking. More studies are needed to decipher the timeline of epithelial barrier impairment and microbiota composition changes during the early course of colitis development.

Previous studies from our laboratory demonstrated that increased bacterial internalization to epithelial cells occurred prior to the onset of TJ damage using mouse models of bowel obstruction and superbug infection [24, 26, 67, 167]. It is believed that upon TJ destruction, luminal bacteria without strain specificity could flow freely through the paracellular space to underlying lamina propria and cause mucosal inflammation. On the other hand, only particular bacterial strains (such as *Escherichia, Staphylococcus, Bacteroides*) have been reported “inside” epithelial cells in our disease models of bowel obstruction and superbug infection [24, 26]. It is possible that the strain-specific bacterial internalization and intracellular survival may act as an initial trigger to evoke damage to paracellular junctional structures, leading to non-specific bacterial translocation and colitis development. The impact of bacterial internalization on epithelial cytoskeletal structures and perijunctional organization has yet to be explored. Furthermore, whether the mucosa-association of bacteria as an early event in transcellular barrier dysfunction may alter the fecal microbiota due to preferential “anchoring” advantage warrants further investigation.

## Common ground hypothesis and further postulation

**Fig. 2:**
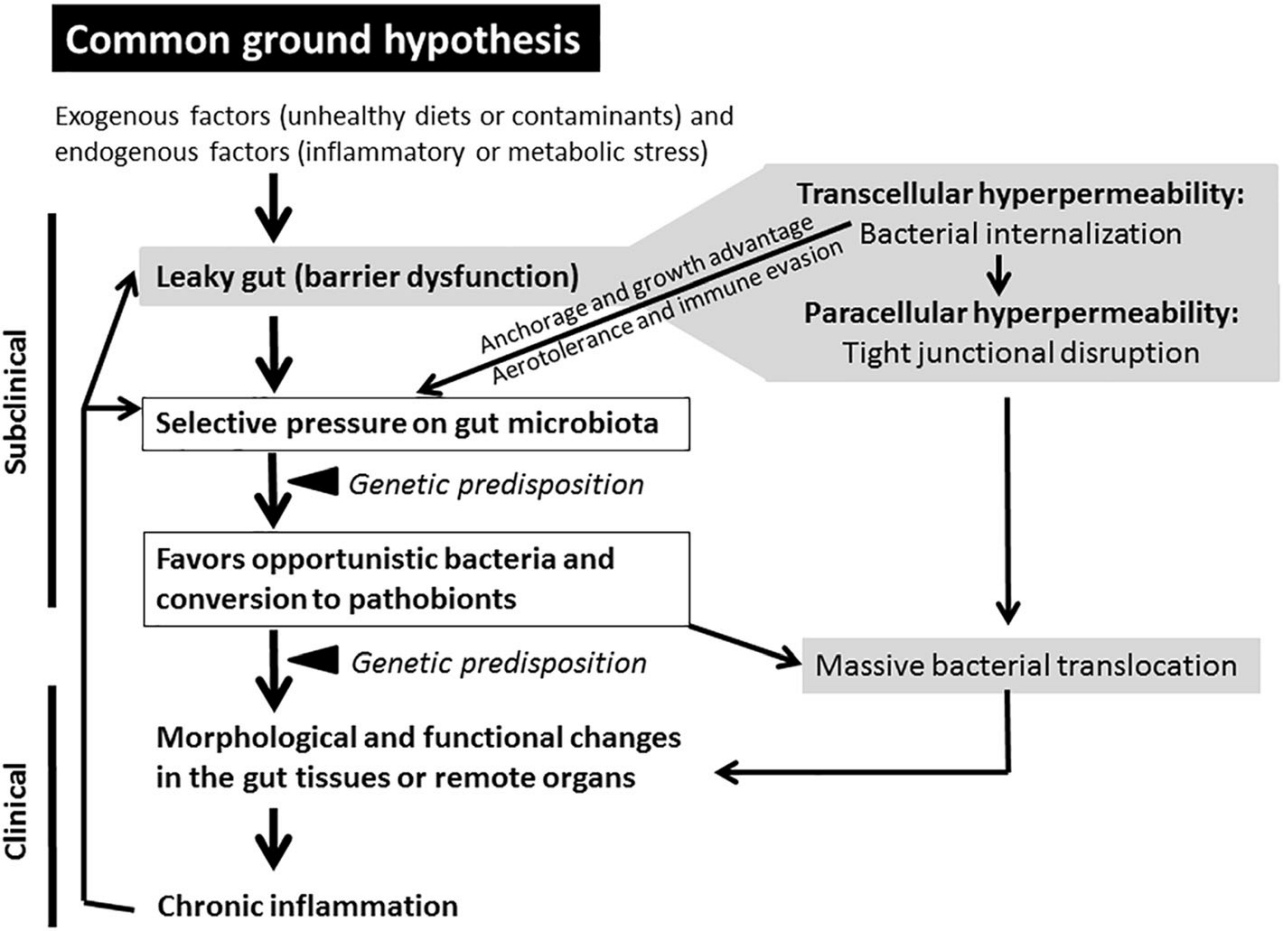
Proposed schema of early pathophysiological changes in epithelial barrier defects and bacterial invasiveness, which causes microbiota dysbiosis and chronic inflammation. The proposed common ground hypothesis depicting the early abnormality of leaky gut that drives microbiota dysbiosis would lead to chronic inflammation. The hypothesis is that endogenous and exogenous factors that trigger gut barrier impairment and low grade immune activation could impose selective pressure on the intestinal microbiota. The subclinical mucosal abnormalities which developed in individuals with genetic predisposition then favor the growth of opportunistic microbes for conversion to pathobionts. The pathobionts subsequently aggravate morphologic and functional changes in gut tissues and remote organs with pathological consequences, and result in chronic inflammation and clinical symptoms. Further postulation with a detailed focus on the gut barriers are added here. We speculate that the initial epithelial barrier dysfunction manifested by transcellular hyperpermeability and passive bacterial internalization may instigate a selection pressure on microbiota (such as positive inforcement by anchorage and growth advantage, and negative impediment by aerotolerance and immune evasion), leading to the emergence of invasive virulent pathobionts. The selection pressure and mucosal pathobionts may cause a shift in the fecal microbial community. On the host’s side, bacterial internalization may also cause epithelial cytoskeletal disorganization and paracellular TJ destruction. The combination of broken epithelial barrier and invasive pathobionts results in a massive amount of bacterial translocation, which leads to clinical features of morphological damage and chronic inflammation. Additional evidence also showed that chronic inflammation may impact on the gut microbiota and cause epithelial death-dependent barrier loss, which eventually leads to vicious cycles of uncontrollable colitis

Disease-predisposing microbiota was found in a wide spectrum of chronic disorders, including IBD and CRC [8–12]. These findings have led to the speculation of a common factor in multigenic disease development. A “common ground hypothesis” was proposed to indicate the key roles of microbiota dysbiosis associated with a leaky gut in the pathogenesis of chronic polygenic diseases [9, 168, 169] (Fig. 2). The hypothesis, which still needs to be rigorously examined, first suggests that endogenous and exogenous factors which cause gut barrier impairment and low grade immune activation could impose selective pressure on the intestinal microbiota. The subclinical mucosal abnormalities that developed in individuals with genetic predisposition then favor the growth of opportunistic microbes with virulence emergence. The opportunistic microbes then aggravate the morphologic and functional changes with pathological consequences, and result in chronic inflammation and clinical symptoms in the host (Fig. 2).

Additional evidence also demonstrated that chronic inflammation may shape the gut microbiota and further contribute to dysbiosis [117, 170]. Several lines of evidence have shown that electron acceptors generated as by-products of the inflammatory responses promoted the outgrowth of facultative anaerobes, such as Enterobacteriaceae [171, 172]. Indeed, the mucosa-associated bacteria have higher oxygen tolerance and catalase expression relative to the fecal dominant species [173], which could be an advantage for microbial competition in the gut ecosystem. Alternatively, proinflammatory cytokines (e.g. IFNγ and TNFα) [24, 67, 164] and opportunistic pathobionts (e.g. AIEC and ETBF) [102, 103, 174] were shown to disrupt epithelial integrity through both transcellular and paracellular pathways. Furthermore, chronic inflammation with high oxidative stress (such as superoxide and nitric oxide) caused epithelial death-dependent barrier loss, which may lead to a vicious cycle of aggravating barrier dysfunction and immune hyperactivation [175, 176].

Based on the current knowledge in intestinal barrier regulation (see review papers [67, 177]), we have reconstructed a more detailed hypothesis in attempt to explain the early interaction between epithelial barriers and microbial conversion. In accordance to the “common ground hypothesis”, we speculated that an initial epithelial barrier dysfunction manifested by a low amount of passive bacterial internalization for enrichment of specific mucosa-associated bacteria was the first event causing an altered microbial community (Fig. 2). The internalized commensal bacteria inside epithelial cells with aerotolerance may acquire virulence factors to ensure survival, immune evasion, and anchoring advantage. The epithelia-associated driving of opportunistic commensals to pathobionts could be a point of no return leading to pathological consequences to the host. Bacterial internalization may also disturb the epithelial cytoskeletal contour and destabilize junctional structures, resulting in the passage of non-specific bacterial strains. The combination of host barrier defects and bacterial invasiveness may evoke a massive amount of bacterial translocation and immune hyperactivation in gut mucosa. The immune hyperactivation could impact on the microbiota and cause a further shift to a disease-promoting microbial composition in individuals with genetic predisposition, which eventually leads to chronic inflammation and malignant transformation (Fig. 2).

To date, our understanding of the role of gut microbiota in human health and disease has been fraught with challenges, partly due to the inability to elucidate this complex host-microbe interaction simply based on patient studies. Irrespective of the orders of host or microbial factors involved in disease progression, the co-existence of microbiota dysbiosis and barrier dysfunction (with reciprocal aggravation) appears to be a common instigator in chronic inflammation. Overall, experimental models evaluting subclinical pathophysiological abnormalities (i.e. microbiota dysbiosis and leaky gut) based on the “common ground hypothesis” may serve as a roadmap to decipher the cause-and-effect relationship of disease mechanisms.

## Unanswered questions and future directions

Despite a consensus exists for the presence of microbiota dysbiosis and barrier disruption, the order of the microbial and host factors in disease pathogenesis has not been established in chronic gut inflammation and colitis-associated CRC. Moreover, mucosa-associated pathobionts have been assumed to derive from unharmful gut commensals, yet without direct evidence. One of the proposed triggers for emergence of pathogenic commensals was the need for adaptation to oxidative stress [178, 179]. Other factors, such as mucosal enrichment and selective pressure, on pathobiont conversion remain to be tested. In addition, the virulence profiles to indicate the conversion of commensal to opportunistic pathobionts still need to be determined. Alternatively, whether virulence factors found in opportunistic bacteria (mostly to confer microbial growth advantage) necessarily indicate pathogenic outcome in the hosts or only to those with genetic deficiency warrant further studies.

Furthermore, the majority of microbiome studies so far have focused on bacterial census, and the roles of virus and fungi are less well understood. Since bacteriophages are transferrable and are abundant in the human gut, their roles in modulating the bacterial ecosystems and conferring opportunistic virulence warrant thorough investigation [180, 181]. In addition, bacterial influx due to transcellular and paracellular hyperpermeability in intestinal epithelia was observed in IBD and CRC patients, yet the relative timing of each pathway remains unclear. Timeline studies in experimental models may answer this question, and will provide insights to the differential regulation of distinct transepithelial routes and their relationships to the shaping of gut microbiota.

Fecal microbiota transplantation (FMT) is now standard of care for recurrent *Clostridium difficile* infection, and emerging evidence also supports the use of FMT to treat IBD [182, 183]. A recent randomized double-blinded controlled trial had shown that FMT induced remission in patients with active UC, which was associated with increased microbial richness without adverse events following transplantation [184, 185]. Some studies reported worsening GI symptoms after FMT in IBD patients by lower GI delivery or in *Clostridium* infection[186], which may be due to variable donor microbial factors. The possible use of restoration of gut barrier as an indicator of colonization of a healthy microbiota following FMT warrant further studies.

## Conclusions

Research for correction of abnormal microbe-host interaction by sealing the broken barrier and improvement of epithelial integrity is currently in progress to supplement anti-inflammatory and immunotherapies for IBD patients [187–189]. Moreover, novel microbe-focused intervention such as bacterial engineering, next-generation probiotics, microbe-specific bactericidal antibiotics, and fecal microbiota transplantation as a monotherapy or add-on therapy will be promising for IBD treatment [190, 191]. Based on the “common ground hypothesis”, targeting the dysbiotic bacteria and intestinal barriers may be used as treatment for not only IBD but also extraintestinal inflammatory disorders and colitis-associated cancers. In addition, the use of microbial signatures in addition to genetic traits as diagnostic biomarkers to predict the prognosis and development of diseases have shown positive results in clinical studies and could be used for personalized medicine in the future [192, 193]. Lastly, diet and prebiotics to affect microbe-microbe and microbe-host interaction would be another valuable approach beyond the known nutritive functions to restore intestinal homeostasis and barrier integrity [194]. In conclusion, the understanding of the core interplay between gut microbiota and host barriers at the early subclinical phase will shed light to novel therapeutic approaches to chronic inflammatory disorders and cancers.

## Abbreviations

AIEC: adherent-invasive *Escherichia coli*
BB: brush border
CD: Crohn’s disease
CEACAM: carcinoemcryonic antigen adhesion molecule
COX: cyclooxygenase
CRC: colorectal carcinoma
DSS: dextran sulfate sodium
ETBF: enterotoxigenic *Bacteroides fragilis*
FMT: fecal microbiota transplantation
IBD: inflammatory bowel disease
MLCK: myosin light chain kinase
NOD: nucleotide-binding oligomerization domain
TJ: tight junction
TLR: toll-like receptor
UC: ulcerative colitis
